# Transarterial infusion chemotherapy combined with lenvatinib and tislelizumab versus intravenous chemotherapy for unresectable pancreatic ductal adenocarcinoma: a retrospective propensity score matching analysis

**DOI:** 10.3389/fonc.2026.1898332

**Published:** 2026-08-19

**Authors:** Haohao Lu, Bin Liang, Qing Fu, Xiangwen Xia

**Affiliations:** 1 Department of Radiology, Union Hospital, Tongji Medical College, Huazhong University of Science and Technology, Wuhan, China; 2 Hubei Province Key Laboratory of Molecular Imaging, Wuhan, China; 3 Hubei Provincial Clinical Research Center for Precision Radiology & Interventional Medicine, Wuhan, China

**Keywords:** lenvatinib, pancreatic ductal adenocarcinoma, propensity score matching, survival prognosis, targeted immunotherapy, tislelizumab, transarterial infusion chemotherapy, unresectable pancreatic cancer

## Abstract

**Background:**

Pancreatic ductal adenocarcinoma (PDAC) remains one of the most lethal malignancies with dismal prognosis for unresectable cases. Systemic chemotherapy shows limited efficacy, while transarterial infusion chemotherapy (HAIC) enables targeted drug delivery, and the combination of anti-angiogenic agents and immune checkpoint inhibitors has demonstrated synergistic effects in solid tumors.

**Methods:**

In this retrospective, single-center propensity score-matched study, 183 unresectable PDAC patients treated between January 2021 and December 2024 were analyzed Propensity score matching (PSM) was performed at a 1:1 ratio, resulting in 45 patients in the HAIC + lenvatinib + tislelizumab group and 45 patients in the intravenous chemotherapy group. The primary endpoints were overall survival (OS) and progression-free survival (PFS); secondary endpoints included objective response rate (ORR), disease control rate (DCR), and safety.

**Results:**

After PSM, baseline characteristics were well-balanced.1). The HAIC + lenvatinib + tislelizumab group achieved significantly higher ORR (37.8% vs. 17.8%, P = 0.023) and DCR (88.9% vs. 64.4%, P = 0.004) than the chemotherapy group. Median OS was 13.1 months (95% CI: 11.4–14.7) in the experimental group versus 8.8 months (95% CI: 7.1–10.9) in the control group (P<0.001). Median PFS was 6.0 months (95% CI: 5.0–6.9) versus 3.7 months (95% CI: 1.9–4.0), respectively (P<0.001). The most common grade ≥3 adverse events were hypertension (15.6% vs. 2.2%, P = 0.012) in the HAIC + lenvatinib + tislelizumab group and neutropenia (24.4% vs. 6.7%, P = 0.008) in the chemotherapy group.

**Conclusions:**

HAIC combined with lenvatinib and tislelizumab significantly was associated with significantly prolonged survival and improved tumor response in unresectable PDAC with manageable toxicity, providing a promising therapeutic strategy for this devastating disease.

## 1 Introduction

### 1.1 Background

Pancreatic ductal adenocarcinoma (PDAC) accounts for over 90% of pancreatic malignancies and ranks as the seventh leading cause of cancer-related deaths worldwide (1). Although serum miRNA signatures (specifically miR-21 and miR-155) as potential non-invasive biomarkers for early detection and operability evaluation, but many patients are already in the advanced stage when they seek medical treatment (2). With its insidious onset and aggressive biological behavior, only 15–20% of patients are eligible for curative surgical resection at diagnosis (3). For the majority of unresectable cases (locally advanced or metastatic), systemic chemotherapy remains the mainstay of treatment. The current first-line regimens, including gemcitabine plus nab-paclitaxel (AG regimen) and FOLFIRINOX, yield modest objective response rates (ORR) of 15–25% and median overall survival (OS) of 8–11 months (4, 5). Despite decades of research, the 5-year survival rate for advanced PDAC remains below 10% (6, 7), highlighting an urgent need for novel therapeutic strategies.

Transarterial infusion chemotherapy (HAIC) has emerged as a promising locoregional treatment for hepatobiliary malignancies, delivering high concentrations of chemotherapeutic agents directly to the tumor vasculature while minimizing systemic exposure (8). Unlike intravenous chemotherapy, HAIC achieves drug concentrations 5–10 times higher in tumor tissues, enhancing cytotoxic effects against poorly vascularized tumors like PDAC (9, 10). Recent studies have extended HAIC application to pancreatic cancer, showing encouraging local control (11, 12). Concurrently, the combination of anti-angiogenic agents and immune checkpoint inhibitors (ICIs) has revolutionized cancer treatment. Lenvatinib, a multi-targeted tyrosine kinase inhibitor (TKI) targeting *VEGFR, FGFR, PDGFR*, and *RET (*13), disrupts tumor angiogenesis and is hypothesized to remodel the tumor microenvironment to enhance ICI efficacy (14–16). Tislelizumab, a humanized anti-PD-1 monoclonal antibody with high affinity for PD-1 and minimal Fcγ receptor binding (17, 18), reduces immune cell exhaustion and restores anti-tumor immunity (19–21).

Preclinical studies have demonstrated synergistic effects of HAIC, anti-angiogenic agents, and ICIs (22–24): HAIC reduces tumor burden and releases tumor-associated antigens, lenvatinib normalizes tumor vasculature to improve immune cell infiltration, and tislelizumab reinvigorates exhausted T cells (25–27). Clinical evidence supporting this triple combination in PDAC is limited, with only a few small-scale studies reporting favorable preliminary outcomes (28–30). To address this gap, we conducted a retrospective study with propensity score matching (PSM) to compare the efficacy and safety of HAIC + lenvatinib + tislelizumab versus standard intravenous chemotherapy in unresectable PDAC.

### 1.2 Research objectives

This study aimed to: (1) Evaluate the efficacy of HAIC combined with lenvatinib and tislelizumab in terms of OS, PFS, ORR, and DCR; (2) Compare the safety profile between the combined regimen and intravenous chemotherapy; (3) Validate the clinical utility of this locoregional-systemic combination strategy for unresectable PDAC.

### 1.3 Hypothesis

We hypothesized that patients receiving HAIC combined with lenvatinib and tislelizumab demonstrated a significant improvement in overall survival compared to those receiving systemic intravenous chemotherapy, with acceptable toxicity profiles in unresectable PDAC patients.

## 2 Materials and methods

### 2.1 Study design and patient selection

This retrospective cohort study was conducted at Union Hospital, Tongji Medical College, Huazhong University of Science and Technology. The study protocol was approved by the Institutional Review Board and conducted in accordance with the Declaration of Helsinki (31). Informed consent was waived due to the retrospective nature.

Eligibility criteria included: (1) Histologically confirmed PDAC (32); (2) Unresectable disease (locally advanced: tumor invasion of major vessels without distant metastasis; metastatic: distant organ involvement) (33); (3) Eastern Cooperative Oncology Group (ECOG) performance status 0–1 (34); (4) Adequate organ function (bilirubin ≤ 1.5 × upper limit of normal [ULN], alanine transaminase/aspartate transaminase ≤ 3 × ULN, creatinine ≤ 1 × ULN, absolute neutrophil count ≥ 1.5 × 10^9^/L, platelet count ≥ 100 × 10^9^/L) (35); (5) No prior systemic chemotherapy for advanced PDAC; (6) Complete clinical and follow-up data.

Exclusion criteria were: (1) Concurrent other malignancies (36); (2) Active infection requiring systemic antibiotics; (3) History of severe cardiovascular disease; (4) Pregnancy or lactation; (5) Known allergy to study drugs; (6) Uncontrolled hypertension (systolic blood pressure > 160 mmHg or diastolic > 100 mmHg).

### 2.2 Treatment protocols

#### 2.2.1 Experimental group: HAIC + lenvatinib + tislelizumab

HAIC was performed via percutaneous femoral artery puncture under local anesthesia. Using digital subtraction angiography (DSA) (37), a 5-French catheter was selectively positioned based on tumor location and vascular supply. For tumors primarily located in the pancreatic head, the gastroduodenal artery (GDA) was preferentially catheterized; for body/tail lesions, the splenic artery or dorsal pancreatic artery branches were targeted. In clinical practice, the celiac artery (CA) served as the primary infusion site in 31 patients (68.9%), while the superior mesenteric artery (SMA) was selected in 14 patients (31.1%), often when SMA branches provided dominant supply to the lesion. For the 20 patients (44.4%) presenting with synchronous liver metastases, priority was given to positioning the catheter in the CA to ensure maximal hepatic arterial coverage, supplemented by adjustments to target the pancreatic tumor bed when feasible. Chemotherapeutic agents included oxaliplatin (85 mg/m^2^, 2-h infusion) and 5-fluorouracil (2400 mg/m^2^, 46-h continuous infusion) plus leucovorin (400 mg/m^2^, 2-h infusion) (FOLFOX regimen) (38). HAIC was repeated every 3 weeks for 4–6 cycles (39).

Oral lenvatinib (8 mg once daily) was initiated 3 days after the first HAIC cycle and continued until disease progression or intolerable toxicity (40). Intravenous tislelizumab (200 mg) was administered every 3 weeks, starting concurrently with the second HAIC cycle, for up to 2 years or until progression (41).

#### 2.2.2 Control group: intravenous chemotherapy

Patients received either AG regimen (gemcitabine 1000 mg/m^2^ on days 1, 8, 15 plus nab-paclitaxel 125 mg/m^2^ on days 1, 8, 15, every 4 weeks) or FOLFIRINOX (oxaliplatin 85 mg/m^2^, irinotecan 180 mg/m^2^, leucovorin 400 mg/m^2^, 5-fluorouracil 400 mg/m^2^ bolus followed by 2400 mg/m^2^ continuous infusion over 46 h, every 2 weeks). Treatment was continued until disease progression, intolerable toxicity, or patient refusal (42).

### 2.3 Data collection

Demographic data (age, gender, BMI), clinical characteristics (ECOG score, tumor location, Tumor stage, presence of liver metastasis, baseline CA19–9 level), treatment details (number of cycles, dose modifications), laboratory results (complete blood count, liver/kidney function), and adverse events (AEs) were extracted from electronic medical records.

Follow-up was conducted every 4 weeks for the first year and every 8 weeks thereafter, including physical examination, laboratory tests, and contrast-enhanced computed tomography (CT) or magnetic resonance imaging (MRI) of the abdomen and pelvis. The last follow-up date was June 30, 2025.

### 2.4 Outcome measures

1. Tumor Response: Assessed every 2 cycles (6 weeks) using RECIST v1.1 criteria (43), categorized as complete response (CR), partial response (PR), stable disease (SD), or progressive disease (PD).
2. ORR: Proportion of patients achieving CR + PR.
3. DCR: Proportion of patients achieving CR + PR + SD.
4. OS: Time from treatment initiation to death or last follow-up.
5. PFS: Time from treatment initiation to disease progression or death.
6. Safety: AEs were graded using CTCAE v5.0 (44), with focus on grade ≥3 events.
7. Tumor response was assessed by two independent, blinded radiologists using RECIST v1.1. Discrepancies were resolved by consensus.

### 2.5 Propensity score matching

PSM was performed to balance baseline confounding factors between groups. A logistic regression model was constructed to calculate propensity scores (PS) using the following covariates: age, gender, BMI, ECOG score, tumor stage (locally advanced vs. metastatic), tumor location (head vs. body/tail), liver metastasis, baseline CA19–9 level (> 37 U/mL vs. ≤ 37 U/mL), and presence of diabetes/hypertension. Patients were matched 1:1 using nearest-neighbor matching with a caliper width of 0.2 × standard deviation of the logit-transformed PS. Matching quality was evaluated by standardized mean differences (SMD), with SMD <0.1 indicating adequate balance (45).

### 2.6 Statistical analysis

Continuous variables were presented as mean ± standard deviation (normal distribution) or median (interquartile range [IQR]) (skewed distribution), compared using independent samples t-test or Mann-Whitney U test. Categorical variables were expressed as counts (percentages), compared using χ^2^ test or Fisher’s exact test. Survival curves were plotted using Kaplan-Meier method and compared with log-rank test. Hazard ratios (HR) and 95% confidence intervals (CI) were calculated using Cox proportional hazards regression. All statistical analyses were performed using R 4.3.1 (R Foundation for Statistical Computing) and SPSS 26.0 (IBM Corp.). Two-sided P < 0.05 was considered statistically significant. To mitigate immortal time bias introduced by the staggered initiation of systemic agents, a landmark analysis was conducted at 6 weeks.

## 3 Results

### 3.1 Patient characteristics before and after PSM

A total of 183 patients were initially enrolled (experimental group: 87, control group: 96). After PSM, 90 patients (45 per group) were included in the final analysis. Baseline characteristics before and after matching are summarized in **Table 1**. Before PSM, the experimental group had a higher proportion of locally advanced disease (47.1% vs. 32.3%, P = 0.028) and lower baseline CA19–9 levels (median: 286 U/mL vs. 412 U/mL, P = 0.015). After PSM, all covariates were well-balanced, confirming successful matching (**Table 1**). Before PSM, 96 patients in the control group received either AG (n=51) or FOLFIRINOX (n=45). After PSM, the distribution remained similar (AG: n=24; FOLFIRINOX: n=21; P = 0.712). Crucially, baseline imbalances persisted in the post-PSM comparison between the experimental group and the FOLFIRINOX subgroup (e.g., a higher proportion of metastatic disease in the FOLFIRINOX cohort, P = 0.041), precluding robust direct subgroup comparisons. The median follow-up was 10.3 months (95% CI: 8.8–11.9), as estimated by the reverse Kaplan-Meier method.

**Table 1:** Baseline characteristics of patients before and after propensity score matching.

Characteristic	Total (n=183)	Experimental group (n=87)	Control group (n=96)	P-value	After PSM (n=90)	Experimental (n=45)	Control (n=45)	SMD
Age, mean ± SD	62.3 ± 8.7	61.8 ± 9.2	62.7 ± 8.3	0.456	62.1 ± 8.9	61.9 ± 9.1	62.3 ± 8.7	0.04
Gender, male, n (%)	109 (59.6)	52 (59.8)	57 (59.4)	0.953	27 (60.0)	27 (60.0)	27 (60.0)	0.00
BMI, mean ± SD	23.5 ± 3.1	23.7 ± 3.3	23.3 ± 2.9	0.381	23.6 ± 3.0	23.7 ± 3.2	23.5 ± 2.8	0.06
ECOG score, 0/1, n (%)	78/105 (42.6/57.4)	41/46 (47.1/52.9)	37/59 (38.5/61.5)	0.218	39/41 (43.3/56.7)	20/25 (44.4/55.6)	19/26 (42.2/57.8)	0.04
Tumor stage, n (%)								
- Locally advanced	65 (35.5)	41 (47.1)	31 (32.3)	0.028	39 (43.3)	20 (44.4)	19 (42.2)	0.05
- Metastatic	118 (64.5)	46 (52.9)	65 (67.7)		51 (56.7)	25 (55.6)	26 (57.8)	0.05
Tumor location, n (%)								
- Head	112 (61.2)	54 (62.1)	58 (60.4)	0.785	55 (61.1)	28 (62.2)	27 (60.0)	0.05
- Body/tail	71 (38.8)	33 (37.9)	38 (39.6)		35 (38.9)	17 (37.8)	18 (40.0)	0.05
Liver metastasis, n (%)	83 (45.4)	38 (43.7)	45 (46.9)	0.642	41 (45.6)	20 (44.4)	21 (46.7)	0.05
Baseline CA19-9, median (IQR), U/mL	348 (126–689)	286 (112–598)	412 (143–765)	0.015	332 (121–654)	328 (118–642)	336 (124–665)	0.02
Diabetes, n (%)	57 (31.1)	25 (28.7)	32 (33.3)	0.478	28 (31.1)	14 (31.1)	14 (31.1)	0.00
Hypertension, n (%)	63 (34.4)	29 (33.3)	34 (35.4)	0.756	30 (33.3)	15 (33.3)	15 (33.3)	0.00

SD, standard deviation; IQR, interquartile range; SMD, standardized mean difference; ECOG, Eastern Cooperative Oncology Group; CA19-9, carbohydrate antigen 19-9.

### 3.2 Treatment compliance

In the experimental group, the median number of HAIC cycles was 4 (range: 3–6), lenvatinib treatment duration was 5.8 months (range: 2.3–11.5), and tislelizumab cycles were 6 (range: 3–12). Dose reductions were required in 13 patients (28.9%): 8 (17.8%) primarily due to lenvatinib-related hypertension, 3 (6.7%) due to tislelizumab-related rash, and 2 (4.4%) due to HAIC-related myelosuppression. Treatment discontinuation due to AEs occurred in 4 patients (8.9%); three of these were attributable to persistent grade 3 hypertension (lenvatinib-related), and one was due to grade 3 pneumonitis (tislelizumab-related). In the control group, the median number of chemotherapy cycles was 4 (range: 2–6), with dose reductions in 16 patients (35.6%): 12 for neutropenia, 3 for thrombocytopenia, and 1 for diarrhea. Treatment discontinuation due to AEs occurred in 4 patients (8.9%) in the experimental group and 5 patients (11.1%) in the control group (P = 0.683).

### 3.3 Efficacy outcomes

#### 3.3.1 Tumor response

After a median follow-up of 10.3 months (IQR: 6.8–14.5), the experimental group achieved significantly higher ORR (37.8% [23.5–54.0]) and DCR (88.9% [75.9–95.3]) than the control group (**Table 2**). Despite the high objective response rate, no patients in the experimental group (0%; 0/45) achieved sufficient downstaging to qualify for curative-intent R0/R1 surgical resection during the follow-up period.

**Table 2:** Tumor response rates by RECIST v1.1 criteria.

Response	Experimental group (n=45), n (%)	Control group (n=45), n (%)	P-value
Complete Response (CR)	2 (4.4)	0 (0.0)	0.497
Partial Response (PR)	15 (33.3)	8 (17.8)	0.086
Stable Disease (SD)	21 (46.7)	21 (46.7)	1.000
Progressive Disease (PD)	7 (15.6)	16 (35.6)	0.019
ORR (CR + PR)	17 (37.8)	8 (17.8)	0.023
DCR (CR + PR + SD)	40 (88.9)	29 (64.4)	0.004

#### 3.3.2 Survival outcomes

**Figure 1:**
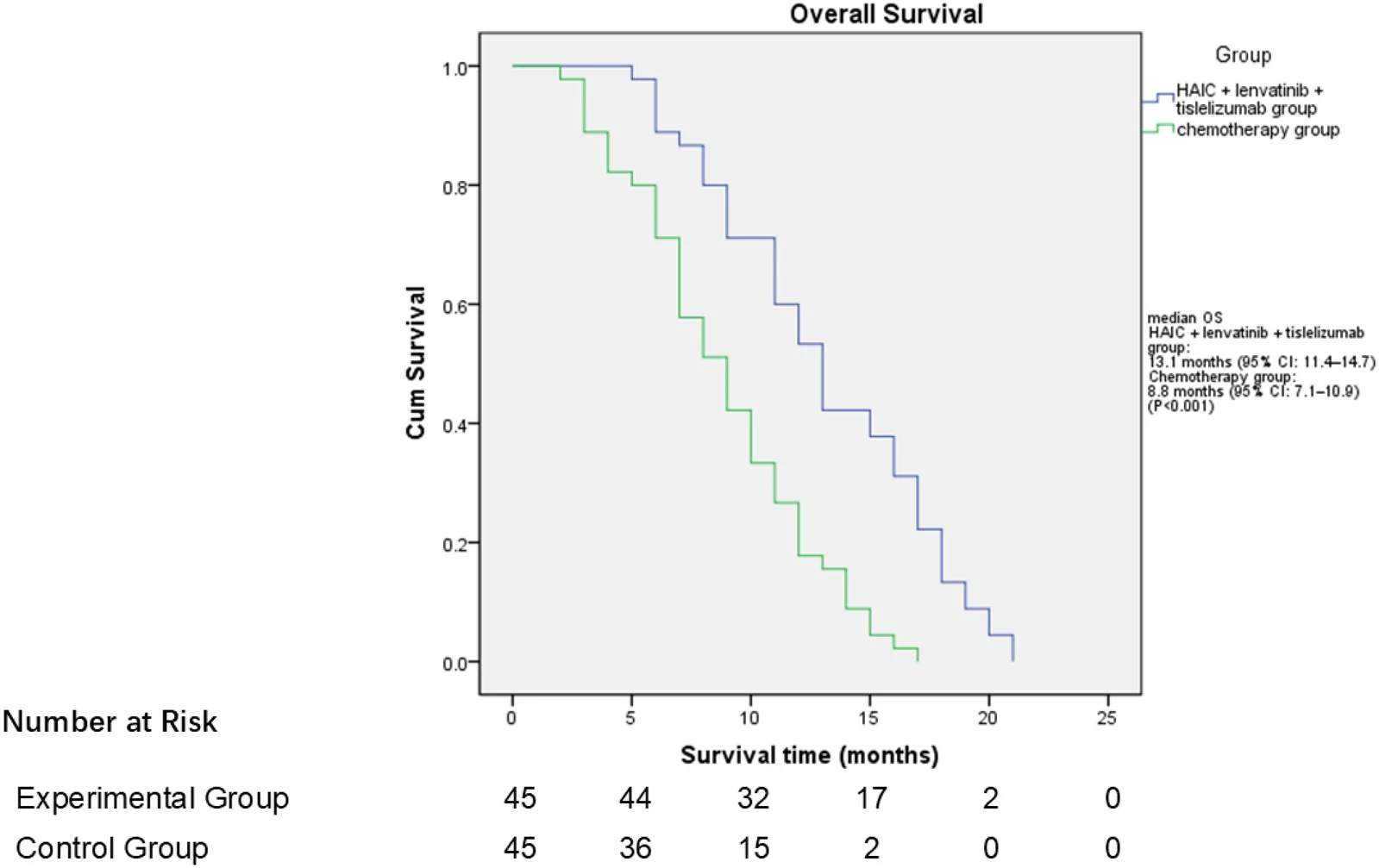
Kaplan-Meier survival curves (overall survival). Kaplan-Meier overall survival (OS) curves for unresectable pancreatic ductal adenocarcinoma (PDAC) patients after 1:1 propensity score matching (PSM). A total of 90 eligible patients were divided into two groups: the experimental group (HAIC + lenvatinib + tislelizumab, n=45) and the control group (standard intravenous chemotherapy, n=45). Log-rank test was used for survival curve comparison, and Cox proportional hazards regression model was applied for multivariate prognostic analysis. The median OS was 13.1 months (95% CI: 11.4–14.7) in the experimental group versus 8.8 months (95% CI: 7.1–10.9) in the control group, with a statistically significant difference (P<0.001). CI, confidence interval; HAIC, transarterial infusion chemotherapy.

**Figure 2:**
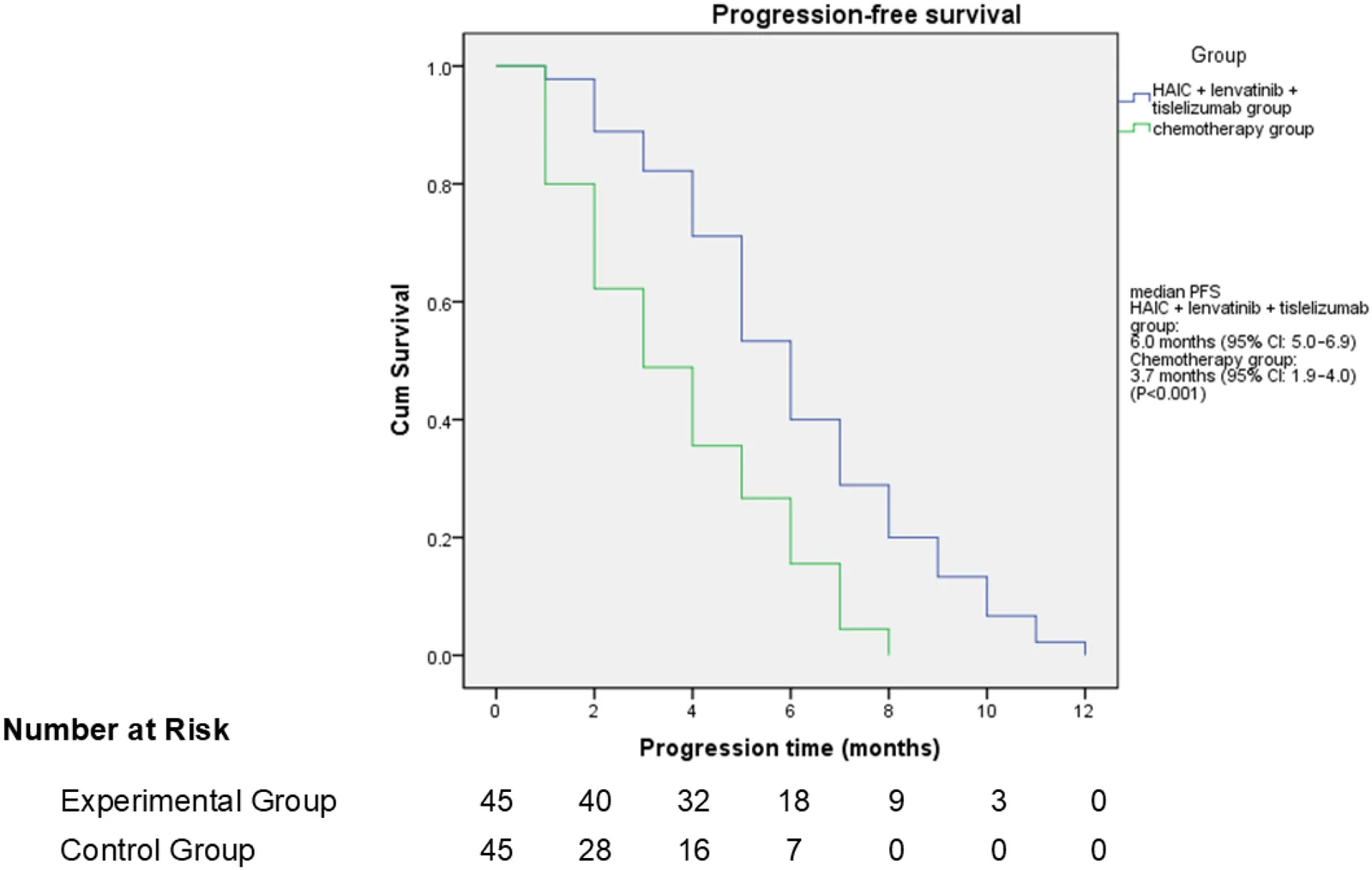
Kaplan-Meier survival curves (progression-free survival). Kaplan-Meier progression-free survival (PFS) curves for PSM-matched unresectable PDAC patients. All enrolled patients received standardized treatment and regular follow-up per study protocol. Survival endpoint was defined as tumor progression confirmed by RECIST v1.1 criteria or all-cause death. The experimental group achieved a significantly prolonged median PFS of 6.0 months (95% CI: 5.0–6.9), compared with 3.7 months (95% CI: 1.9–4.0) in the intravenous chemotherapy control group (P<0.001). RECIST, Response Evaluation Criteria in Solid Tumors.

Median OS was significantly longer in the experimental group (13.1 months, 95% CI: 11.4–14.7) than in the control group (8.8 months,95% CI: 7.1–10.9) (P<0.001, **Figure 1**). Median PFS was 6.0 months (95% CI: 5.0–6.9) in the experimental group versus 3.7 months (95% CI: 1.9–4.0) in the control group (P<0.001, **Figure 2**). Subgroup analysis showed consistent survival benefits across subgroups of tumor stage, liver metastasis, and baseline CA19–9 level (**Table 3**). In a multivariable Cox regression model adjusting for age, ECOG status, tumor stage, liver metastasis, baseline CA19-9, and chemotherapy regimen, the experimental group remained an independent predictor of improved OS (adjusted HR: 0.43, 95% CI: 0.26–0.71, P = 0.001) (**Table 4**).

**Table 3:** Subgroup analysis of overall survival.

Subgroup	Experimental group (n=45)	Control group (n=45)	HR (95% CI)	P-value
Tumor stage				
- Locally advanced	13.2 (11.5–14.9)	8.9 (7.6–10.2)	0.39 (0.20–0.76)	0.005
- Metastatic	10.5 (8.8–12.2)	6.8 (5.9–7.7)	0.47 (0.27–0.82)	0.008
Liver metastasis				
- Yes	10.1 (8.3–11.9)	6.2 (5.3–7.1)	0.41 (0.23–0.73)	0.002
- No	12.8 (11.0–14.6)	8.5 (7.3–9.7)	0.45 (0.24–0.85)	0.013
Baseline CA19-9				
- ≤ 37 U/mL	14.5 (12.1–16.9)	10.2 (8.4–12.0)	0.35 (0.15–0.82)	0.016
- > 37 U/mL	11.2 (9.6–12.8)	7.2 (6.3–8.1)	0.46 (0.28–0.75)	0.002

HR, hazard ratio; CI, confidence interval.

**Table 4:** Multivariable cox regression analysis for survival outcomes.

Variable	OS			PFS		
	aHR	95% CI	P	aHR	95% CI	P
Treatment (Exp vs. Ctrl)	0.43	0.26–0.71	0.001	0.47	0.29–0.76	0.002
ECOG PS (1 vs. 0)	1.48	0.91–2.41	0.115	1.45	0.90–2.33	0.128
Stage (Met. vs. LA)	1.72	1.05–2.82	0.031	1.63	1.01–2.63	0.047
Liver Mets (Yes vs. No)	1.65	1.01–2.70	0.046	1.58	0.98–2.55	0.061
CA19-9 (>ULN vs. ≤ULN)	1.98	1.21–3.24	0.006	1.91	1.18–3.09	0.008
Chemo Regimen (FFX vs. AG)	0.85	0.53–1.36	0.498	0.82	0.52–1.29	0.395

aHR, adjusted hazard ratio; OS, overall survival; PFS, progression-free survival; Exp, Experimental (TAIC+Lenvatinib+Tislelizumab); Ctrl, Control; ECOG PS, Eastern Cooperative Oncology Group Performance Status; Met, Metastatic; LA, Locally Advanced; FFX, FOLFIRINOX; AG, Gemcitabine + nab-Paclitaxel.

### 3.4 Safety analysis

All patients experienced at least one adverse event (**Table 5**). The most common grade ≥3 AEs in the experimental group were hypertension (15.6%), hand-foot skin reaction (6.7%), and proteinuria (4.4%). In the control group, neutropenia (24.4%), thrombocytopenia (13.3%), and febrile neutropenia (6.7%) were the leading grade ≥3 AEs. Immune-related adverse events (irAEs) in the experimental group included hypothyroidism (20.0%), rash (13.3%), and pneumonitis (2.2%), all of which were manageable with symptomatic treatment or temporary drug interruption. No treatment-related deaths occurred in either group. The relative dose intensity for lenvatinib was 78.2%. Post-progression, 22 patients (48.9%) in the experimental group and 25 (55.6%) in the control group received second-line therapy (primarily liposomal irinotecan-based regimens).

**Table 5:** Adverse events (grade ≥3) by CTCAE v5.0.

Adverse event	Experimental group (n=45), n (%)	Control group (n=45), n (%)	P-value
Hematologic			
- Neutropenia	3 (6.7)	11 (24.4)	0.008
- Thrombocytopenia	2 (4.4)	6 (13.3)	0.115
- Anemia	1 (2.2)	3 (6.7)	0.365
- Febrile neutropenia	0 (0.0)	3 (6.7)	0.118
Gastrointestinal			
- Nausea/vomiting	2 (4.4)	4 (8.9)	0.453
- Diarrhea	1 (2.2)	3 (6.7)	0.365
- Abdominal pain	3 (6.7)	2 (4.4)	0.683
Dermatologic			
- Hand-foot skin reaction	3 (6.7)	0 (0.0)	0.071
- Rash	2 (4.4)	1 (2.2)	0.621
Cardiovascular			
- Hypertension	7 (15.6)	1 (2.2)	0.012
- Proteinuria	2 (4.4)	0 (0.0)	0.497
Hepatic			
- Elevated transaminases	2 (4.4)	3 (6.7)	0.683
Immune-related			
- Hypothyroidism	Grade 1-2: 18 (40.0%), Grade ≥3: 9 (20.0%)	0 (0.0)	001
- Rash	Grade 1-2: 10 (22.2%), Grade ≥3: 6 (13.3%)	1 (2.2)	0.039
- Pneumonitis	Grade 1-2: 3 (6.7%), Grade ≥3: 1 (2.2%)	0 (0.0)	0.497

CTCAE: Common Terminology Criteria for Adverse Events.

## 4 Discussion

### 4.1 Key findings

This retrospective PSM study demonstrated that HAIC combined with lenvatinib and tislelizumab significantly improved survival outcomes and tumor response in unresectable PDAC compared to standard intravenous chemotherapy. The experimental group achieved a median OS of 13.1 months and PFS of 6.0 months, representing a 4.3-month and 2.3-month improvement, respectively, over the control group. Additionally, ORR (37.8% vs. 17.8%) and DCR (88.9% vs. 64.4%) were substantially higher in the combined regimen, with manageable toxicity (46). These results support the clinical utility of this triple combination strategy for unresectable PDAC (**Table 6**).

**Table 6:** Comparative summary of therapeutic strategies for unresectable PDAC.

Therapeutic regimen	Biological rationale	Current clinical evidence	Key advantages	Existing limitations
HAIC + Lenvatinib + Tislelizumab (Experimental Regimen)	HAIC achieves local high-concentration chemotherapy; lenvatinib normalizes tumor vasculature and remodels TME; tislelizumab activates anti-tumor immunity; triple-modal synergistic anti-tumor effect	13.1-month median OS, 6.0-month median PFS, 37.8% ORR, 88.9% DCR; low severe hematological toxicity; manageable irAEs (current study)	Superior survival and tumor control efficacy; reduced systemic chemotherapy toxicity; dual local and systemic tumor suppression; good clinical feasibility	Requires professional interventional techniques; partial patients suffer from lenvatinib-induced hypertension; lack of prospective multi-center verification
Standard Intravenous Chemotherapy (AG/FOLFIRINOX)	Systemic cytotoxic chemotherapy kills proliferative tumor cells; single systemic anti-tumor mechanism without targeted immune regulation	8.5–11.1-month median OS, 3.7-month median PFS, 15%–25% ORR; high incidence of severe neutropenia and thrombocytopenia	Mature and standardized clinical regimen; no special technical requirements; wide clinical applicability	Poor local tumor control; severe systemic hematological toxicity; limited survival benefit; easy to induce tumor drug resistance

### 4.2 Comparison with previous studies

Findings from the 2022 Global Cancer Statistics reveal 511,000 incident cases and 467,000 fatalities attributed to pancreatic cancer worldwide, ranking it as the 12th most prevalent cancer in terms of incidence and the 6th leading cause of cancer-related mortality globally. Given that the majority of patients are diagnosed at an advanced clinical stage, the 5-year survival rate is estimated to be merely around 12% (47). Systemic chemotherapy constitutes the mainstay of first-line therapeutic strategies for metastatic pancreatic ductal adenocarcinoma (PDAC), with three pivotal regimens demonstrating distinct median overall survival (OS) profiles: gemcitabine plus nab-paclitaxel (8.5 months) (5), FOLFIRINOX (a combination of irinotecan, leucovorin, fluorouracil, and oxaliplatin; 11.1 months) (48), and NALIRIFOX (folinic acid, liposomal irinotecan, fluorouracil, and oxaliplatin; 11.1 months) (49). Additionally, immunotherapeutic efficacy remains suboptimal in the vast majority of patients, with only a minor subset (approximately 1%) harboring microsatellite instability-high (MSI-high)/mismatch repair-deficient (dMMR) phenotypes deriving clinical benefit (50). For instance, monotherapy with durvalumab, durvalumab in conjunction with tremelimumab, and nivolumab combined with chemotherapeutic agents have all exhibited limited therapeutic responses in unselected patient populations (51, 52).

Our findings align with recent evidence supporting the synergy between locoregional therapy, anti-angiogenics, and ICIs (53). A phase II study by Huizi Sha et al. (54) evaluated penpulimab and anlotinib with nab-paclitaxel/gemcitabine in metastatic pancreatic cancer, reporting a median OS of 13.7 months and median PFS of 8.8 months. Similarly, a case series of lenvatinib-based combinations showed promising local control in unresectable PDAC (55). In contrast, standard intravenous chemotherapy regimens yield median OS of 8.5–11.1 months (5, 48, 49), and dual ICI + TKI combinations (without HAIC) have shown modest ORR of 20–25% (56, 57). The addition of HAIC in our study likely enhanced local drug delivery, overcoming the poor vascularization and dense desmoplastic stroma that limit systemic therapy efficacy in PDAC (58).

### 4.3 Mechanistic insights

Immunotherapeutic strategies have demonstrated modest therapeutic efficacy in PDAC, primarily due to the unique biological features of the tumor itself and its associated tumor microenvironment (TME) (59). Key characteristics of the PDAC TME include a desmoplastic and fibrotic stromal compartment, coupled with reduced vascular density—pathological changes that culminate in elevated interstitial fluid pressure (IFP), impaired tumor perfusion, intratumoral hypoxia, an immunosuppressive milieu, and inadequate bioavailability of systemic therapeutic agents (56, 60). Targeting the compromised vascular phenotype—characterized by immature, dysfunctional blood vessels—may represent a promising therapeutic strategy for PDAC. In an orthotopic murine model of PDAC, vascular normalization—marked by enhanced pericyte investment in vascular structures and ameliorated tumor perfusion—resulted in attenuated intratumoral hypoxia and improved delivery of chemotherapeutic drugs (61). Furthermore, administration of low-dose anti-angiogenic agents to normalize the vasculature can mitigate immunosuppression by dampening the functional activity of regulatory T (Treg) cells and myeloid-derived suppressor cells (MDSCs). This vascular-modulating approach, in turn, facilitates T cell activation and promotes their intratumoral infiltration (62, 63).

Based on preclinical evidence, we hypothesize that the observed clinical benefits of the triple combination may be attributed to several potential synergistic mechanisms First, HAIC delivers high concentrations of chemotherapeutic agents directly to the tumor, inducing tumor cell death and releasing tumor-associated antigens (TAAs) (64, 65). Second, lenvatinib normalizes the tumor vasculature by inhibiting VEGFR signaling, reducing interstitial fluid pressure and improving T cell infiltration (66–68). Third, tislelizumab blocks the PD-1/PD-L1 pathway, reinvigorating exhausted T cells primed by TAAs (69–71). Additionally, lenvatinib may further enhance anti-tumor immunity by reducing myeloid-derived suppressor cells and regulatory T cells (72). This “triple hit” strategy addresses the key barriers to PDAC treatment: poor drug delivery, immune desert microenvironment, and therapeutic resistance (73). While Tao Qin et al. (47) demonstrated that SMAD2/3 signaling mediates perineural invasion—a key driver of PDAC morbidity—our regimen’s anti-VEGF component likely complements this by normalizing aberrant vessels, thereby enhancing T-cell infiltration. However, the retrospective nature of our study precludes definitive conclusions regarding biomarker selection; future work should integrate liquid biopsy profiles, such as the miRNA signatures proposed by Qiuliang Yan et al. (2), to refine patient selection for this triplet regimen.

### 4.4 Safety considerations

The safety profile of the combined regimen was manageable, with no unexpected toxicities. Hypertension and hypothyroidism were the most common grade ≥3 AEs in the experimental group, consistent with previous reports of lenvatinib and tislelizumab combinations (28, 74, 75). Notably, hematologic toxicities (neutropenia, thrombocytopenia) were significantly less frequent in the experimental group, likely due to the locoregional nature of HAIC reducing systemic chemotherapy exposure (76–79). The low rate of severe irAEs (2.2% pneumonitis) confirms the safety of combining HAIC with immunotherapy, as locoregional therapy may mitigate systemic immune-related toxicity (80–82).

### 4.5 Limitations

Several limitations warrant consideration. First, the retrospective, single-center design introduces inherent selection biases that PSM cannot fully eradicate. Notably, we lacked data on molecular subtypes (e.g., *KRAS* mutation status, *BRCA/HRD*) and detailed radiologic scores of vascular invasion, which likely represent residual confounding. Second, the control group comprised two distinct regimens (AG and FOLFIRINOX); while we adjusted for this in multivariable models, the sample size precluded robust subgroup survival comparisons. Third, the sequential administration of HAIC followed by systemic agents introduces potential immortal time bias, although a 6-week landmark analysis supported our findings. Fourth, the absence of biomarker analyses (PD-L1, tumor mutational burden/TMB, microsatellite instability/MSI) limits our ability to identify predictive responders. Finally, the technical expertise required for TAIC limits the immediate generalizability of this approach to community hospitals without dedicated interventional radiology expertise.

### 4.6 Clinical implications

Our results provide a new therapeutic option for unresectable PDAC, particularly for patients with locally advanced disease or liver metastasis who have limited treatment options. The combined regimen offers a favorable benefit-risk ratio, with significant survival improvement and reduced hematologic toxicity compared to standard chemotherapy. Future directions include prospective multi-center trials to validate these findings, exploring predictive biomarkers, and optimizing treatment sequencing (e.g., HAIC followed by maintenance lenvatinib + tislelizumab). The generalizability of this approach is currently limited by the technical expertise required for selective catheterization of pancreatic arteries and the lack of standardization across institutions. Widespread adoption would necessitate specialized training and careful patient selection in high-volume centers.

### 4.7 Limitations of research hypothesis and further experimental validation directions

Although our proposed triple-synergy mechanism hypothesis is supported by preclinical studies and clinical outcome data, there are still certain limitations. First, the current synergistic mechanism inference is based on population-level clinical data and indirect preclinical evidence, lacking direct experimental verification of patient-derived PDAC tumor tissues. Second, the optimal intervention timing, drug dosage gradient, and treatment cycle of HAIC combined with targeted immunotherapy have not been quantitatively verified, and the dose-effect relationship remains unclear. Third, the regulatory effect of the triple-combination regimen on PDAC tumor immune microenvironment and stromal fibrosis lacks in-depth molecular mechanism verification.

Further experimental and clinical research is required in the future: (1) Collect patient tumor specimens to detect changes in immune cell infiltration, cytokine expression and stromal fibrosis before and after treatment, to clarify the *in vivo* synergistic mechanism; (2) Establish PDAC animal models to verify the anti-tumor effect and optimal administration scheme of the triple-combination regimen; (3) Explore molecular biomarkers to guide individualized precise treatment.

## 5 Conclusions

In conclusion, HAIC combined with lenvatinib and tislelizumab significantly improves OS, PFS, ORR, and DCR in unresectable PDAC with manageable toxicity. This locoregional-systemic combination strategy addresses the unique challenges of PDAC treatment and provides a promising new therapeutic avenue for this devastating disease. Prospective studies are warranted to confirm these findings and establish this regimen as a standard of care for unresectable PDAC.

## Data Availability

The raw data supporting the conclusions of this article will be made available by the authors, without undue reservation.
